# Therapeutic plasma exchange as a routine therapy in septic shock and as an experimental treatment for COVID-19: we are not sure

**DOI:** 10.1186/s13054-020-02943-1

**Published:** 2020-05-15

**Authors:** Patrick M. Honore, Aude Mugisha, Luc Kugener, Sebastien Redant, Rachid Attou, Andrea Gallerani, David De Bels

**Affiliations:** ICU Department, Centre Hospitalier Universitaire Brugmann-Brugmann University Hospital, Place Van Gehuchtenplein, 4, 1020 Brussels, Belgium

We read with interest the recent editorial by Keith et al. who concluded that their practice has changed based on their experience, and they now often utilize therapeutic plasma exchange (TPE) earlier in the clinical course of septic shock with multiple organ failure (MODS) and acute respiratory distress syndrome (ARDS) rather than using it as a “rescue therapy” [1]. We would like to make some comments. They quoted several studies, including one from Knaup et al., in order to support their argument [2]. They assert that TPE is unique by offering benefit on multiple levels by removing inflammatory cytokines, stabilizing endothelial membranes, and resetting the hypercoagulable state [1]. Knaup et al. stated that a major difference between TPE and modern extracorporeal adsorption strategies is based on the fact that the exchange of septic shock plasma with fresh frozen plasma may not lead to an unselective depletion of pro- and anti-inflammatory cytokines and will rather replenish protective factors (within FFPs) that have been consumed by the sepsis [2]. It is currently impossible when employing an unselective removal technique to know if we are doing something good by removing an excess of pro-inflammatory mediators or something wrong by removing anti-inflammatory mediators. At this time, we are unable to clearly identify at the bedside which patients are in a pro-inflammatory state that could kill them or an anti-inflammatory state that could help them to survive. The fact that the inflammation is huge during COVID-19 does not justify the non-selective removal of inflammation components, when some elements may be saving patients. TPE also has the potential to cause harm by diluting or attenuating the host’s adaptive response to infection by depletion of immunoglobulins and complement component 3 and 4 in individuals treated with plasmapheresis [3]. Importantly, in the case of SARS-CoV-2 outbreak, TPE will remove the protective antibodies formed by the patient, which is not desirable. In conclusion, TPE may not restore immune homeostasis but may rather aggravate immunoparalysis [4]. We agree with the authors that this outbreak should serve as an impetus to investigate therapies targeting the pathways that lead to morbidity and mortality in these syndromes [1]. This does not mean that we have received a signed blank check to start a therapy without deeply reviewing the rationale and the quality of existing data.

## Authors’ response

Philip Keith, Matthew Day, Linda Perkins, Lou Moyer, Kristi Hewitt and Adam Wells

We appreciate Dr. Honore’s interest and his insight, and we share his concerns regarding the need to be cautious. The points made in the letter are valid and warrant further investigation. The intent of our editorial was not to protocolize the use of TPE in sepsis and/or COVID-19, rather to highlight its potential and to encourage further study [1].

The COVID pandemic has been an eye-opening and humbling experience. Currently, there are no proven treatments for this disease. A number of therapies have been/are being investigated, but early results have been disappointing. Critically ill patients with COVID continue to die at an alarming rate—a recent case series published in JAMA reported 88.1% mortality for patients requiring mechanical ventilation in a large health care system in New York [5].

Altering the host immune response certainly comes with risk, and most patients who survive do so without direct alteration of this pathway. However, when “dysregulated,” this response may lead to refractory hypotension with shock, multiple organ failure, ARDS, and/or death. Therapies targeting specific components continue to be explored, but none have proven to be efficacious thus far (including during the current COVID pandemic). Readily available laboratory testing has not allowed for identification of patients likely to benefit (or those likely to worsen). At this time, in the absence of more specific labs, the clinician’s challenge of evaluating patients and making the most appropriate treatment decisions must mainly be guided by the clinical parameters, as determined by currently available resources and evidence.

We submitted our editorial because of enthusiasm for TPE as a potential supportive and adjunctive treatment for select critically ill, septic patients. We agree to suggest its promotion as a panacea, deserving of a blank check, would be a gross overstatement. TPE has not been appropriately studied, despite decades of evidence suggesting a potential benefit. We feel that the medical community has a responsibility to investigate its role as adjunctive therapy, through well-designed clinical trials.

## Data Availability

Not applicable.

## Abbreviations

MODS: Multi-organ dysfunction syndrome
ARDS: Acute respiratory distress syndrome
TPE: Therapeutic plasmapheresis
FFP: Fresh frozen plasma

## References

[1] Keith P, Day M, Perkins L, Moyer L, Hewitt K, Adam W (2020). A novel treatment approach to the novel coronavirus: an argument for the use of therapeutic plasma exchange for fulminant COVID-19. Crit Care.

[2] Knaup H, Stahl K, Schmidt BMW, TO I, Busch M, Wiesner O (2018). Early therapeutic plasma exchange in septic shock: a prospective open-label nonrandomized pilot study focusing on safety, hemodynamics, vascular barrier function, and biologic markers. Crit Care.

[3] Rimmer E, Houston BL, Kumar A, Abou-Setta AM, Friesen C, Marshall JC (2014). The efficacy and safety of plasma exchange in patients with sepsis and septic shock: a systematic review and meta-analysis. Crit Care.

[4] Szczeklik W, Wawrzycka K, Włudarczyk A, Sega A, Nowak I, Seczyńska B (2013). Complications in patients treated with plasmapheresis in the intensive care unit. Anaesthesiol Intensive Ther.

[5] Richardson S, Hirsch JS, Narasimhan M, et al. Presenting characteristics, comorbidities, and outcomes among 5700 patients hospitalized with COVID-19 in the New York City area. JAMA Published online April 22. 2020. 10.1001/jama.2020.6775. 10.1001/jama.2020.6775 PMC7177629 32320003

